# Editorial: Insights in consciousness research, volume II

**DOI:** 10.3389/fpsyg.2025.1602845

**Published:** 2025-05-07

**Authors:** Monia D'Angiò, Luca Simione, Xerxes D. Arsiwalla, Antonino Raffone

**Affiliations:** ^1^Department of Psychology, Sapienza University of Rome, Rome, Italy; ^2^Institute of Cognitive Sciences and Technologies, Consiglio Nazionale delle Ricerche, Rome, Italy; ^3^Faculty of Interpreting and Translation, UNINT, Università degli Studi Internazionali, Rome, Italy; ^4^Wolfram Institute for Computational Foundations of Science, Champaign, IL, United States

**Keywords:** consciousness, non-dual awareness, phenomenology, minimal self, artificial intelligence, pain

Advancing upon the scientific program of the inaugural Research Topics in this series on insights and rising stars in consciousness research (Arsiwalla et al., 2023; Srinivasan et al., 2023), this second edition seeks to explore classic debates in consciousness science, such as distinguishing between the most promising contemporary theories of consciousness, while also offering fresh perspectives and new insights into the progress of this field, including current reflections on its connection to artificial intelligence.

One of the most debated issues in consciousness research concerns its neural correlates (NCCs). Although researchers often aim to distinguish proper NCCs from their prerequisites and consequences (Aru et al., 2012; Seth and Bayne, 2022), new approaches are being developed in the field. Fink proposed a framework based on direct neurophenomenal structuralism, which directly relates neural structures to the structures of phenomenal experience without postulating intermediate levels of explanation. To achieve this, the author introduced a classification of four “sufficiency tests” designed to determine which systems are conscious (Which-test), when they are conscious (When-test), their conscious content (What-test), and how they are phenomenally experienced (How-test). According to the author, the How-test is best approached through direct neurophenomenal structuralism. These methodologies should guide experimental investigations of consciousness and the formulation of hypotheses regarding NCCs.

In the same vein, Josipovic argues that conscious awareness does not require the mediation of mental representations. As such, a dedicated network, distinct from the neural correlates of cognitive processing, should account for the dynamics of consciousness. In his theory of the reflexivity gradient of consciousness, Josipovic highlights that consciousness research predominantly investigates its phenomenal aspects, such as content, arousal level, and cognitive processing, often neglecting consciousness itself. This non-dual awareness, with its inherent, non-representational reflexivity, is characterized by an implicit-explicit gradient of experience that is independent of both the content of experience and the state of experiencing.

Pehlivanova et al. conducted an original study to test whether different cognitive styles [i.e., actively open-minded thinking (AOT) and need for closure (NFC)] influence how psychic researchers, compared to academics from other disciplines and lay believers, evaluate data on such phenomenological experiences. Results showed that psychic academics exhibit a level of AOT similar to that of other academics, with both groups differing from lay believers. This demonstrates that psychic researchers possess strong critical thinking skills and are not biased in their engagement with research on psychic phenomena.

Qualitative phenomenology is a cross-disciplinary methodology that has been applied in various fields of study. An interesting application can be found in clinical psychiatric research. Oblak et al. conducted a single-case study of a patient with psychiatric comorbidities, collecting data over 2 years to construct a personalized network model (PNM) explaining psychiatric disorders within a phenomenology-informed framework. By incorporating various measures, including phenomenological, neuropsychological, and language assessments, the resulting PNM identified a core maladaptive pattern of sensemaking and disorders of self described as “the crisis of objectivity.” These data demonstrate that PNM can be effectively incorporated into qualitative phenomenological methods applied to clinical psychiatric research.

Another aspect related to qualia (phenomenal experience) is the minimal self, a first-person, pre-reflective self-awareness. Gallagher proposed that the minimal self is linked to both the sense of ownership and the sense of agency, which pertains not only to bodily actions but also extends to cognitive processes such as thinking and imagining—implying that we are the agents of our own cognition. Similarly, the sense of ownership is not limited to bodily ownership alone. However, in everyday life, directly perceiving minimal experience can be challenging. Only specific phenomenal practices, such as meditation, sensory deprivation, or experimental conditions, can provide insights into the experience of the minimal self.

A framework to investigate the qualitative aspects of consciousness was established by Tsuchiya et al., utilizing quantum theory to formulate the Quantum-like Qualia (QQ) hypothesis. Traditionally, qualia are treated as fixed points in a dimensional space, assuming they can be measured without alteration. However, empirical evidence suggests that internal attention can modify qualia during measurement. In this model, qualia, encompassing all possible aspects of experience, are referred to as “observables,” while sensory inputs and internal conditions (e.g., attention) are considered “states” that influence “measurement outcomes,” resulting from their interaction. The predictions of the QQ hypothesis align with experimental findings, offering new perspectives on the relationship between consciousness and attention.

According to Andersen, some aspects, such as evolutionary biology, Occam's Razor, and Hume's Dilemma, are often overlooked or inadequately addressed in existing models of consciousness. In an attempt to incorporate these aspects, the author proposed the Maps of Meaning theory of consciousness, which is grounded in a first-principles approach to defining consciousness and integrate psychology, neuroscience, religion, and philosophy. In this theory, consciousness is conceptualized as the inevitable byproduct of having multiple goals and the continuous process of evaluating and prioritizing these goals to guide action in the world.

Instead of introducing new theories of consciousness, some authors have focused on models for evaluating and distinguishing existing theories (Kirkeby-Hinrup) or integrating them (Ruan).

Kirkeby-Hinrup proposed a methodological framework to better explain and quantify the evidence supporting theories of consciousness. Two approaches are currently used in the literature: (1) collaboration between proponents of different theories to develop paradigms that test their respective predictions (ARC; e.g., Consortium et al., 2023; Melloni et al., 2023); and (2) the establishment of a set of criteria to assess the scope and explanatory power of each theory regarding conscious phenomena, largely independent of empirical data (CRIT; Doerig et al., 2021). Building on these two approaches, the author introduced the “quantification to the best explanation” (QBE) method, based on Bayesian confirmation theory, to complement and address the shortcomings of the existing approaches.

Ruan proposed an integrative approach aimed at unifying existing theories of consciousness. In this process, two key aspects must be considered: first, ensuring that the theories being examined genuinely address consciousness itself by properly defining different global states of consciousness; second, critically evaluating the methods and strategies used to study consciousness. Instead of merely attempting to unify theories of consciousness (ToCs), the author proposed a layered architecture of the mind as a potential way to reconcile even competing theories. In this model, multiple signals are processed simultaneously, involving several brain regions and mechanisms. The formation of multiple, temporary zones of consciousness, which can be arbitrarily bounded, results in experiences with specific and distinct attributes.

Due to advancements in artificial intelligence (AI) technology, another debated issue in consciousness science is whether AI could exhibit conscious properties. Prentner and Hoffman offered insights on the potential inclusion of AI within a framework of consciousness. Their approach is based on the conscious agent theory (CAT; Hoffman and Prakash, 2014), which relies on rigorous mathematical assumptions and emphasizes the fundamental role of agency in selecting a particular experience from a set of possible experiences, making it probabilistically measurable. In this view, experience itself constitutes the first-person aspect of consciousness, while its consequences are what can be observed and measured. Alongside CAT, the interface theory of perception (ITP; Hoffman et al., 2015) conceptualizes perception as a kind of interface with the world, enabling an agent to interact with reality. Within this framework, consciousness is understood as a network of conscious agents that represent themselves through interfaces, forming a self-reflective, non-dual awareness.

Building on reflections about AI, Mogi explores the potential computational role of consciousness as an alternative approach to studying consciousness beyond phenomenology. While several cognitive functions, such as attention regulation, adaptation to new contexts, and embodied cognition, may be uniquely associated with conscious processing, it remains unclear which computations are specifically tied to consciousness. Moreover, the study introduces the concept of “conscious supremacy”—inspired by quantum supremacy—to distinguish computations that require consciousness from those that can be performed unconsciously.

Like consciousness, pain is a complex state involving a range of qualia and psychological (cognitive) processes. Gray Hardcastle offers an insightful perspective on studying pain, suggesting that pain, rather than being localized to a single brain region, emerges from a widespread activation pattern that partially overlaps with other sensory and cognitive processes. From a connectivity-based perspective, multiple brain areas contribute to various functions rather than operating in isolation. Given the heterogeneity of neuronal responses, also the experience of pain—like consciousness—might be dynamic and adaptive, shaped by shifting patterns of brain activity over time, rather than being reducible to fixed neural mechanisms.

The articles included in this Research Topic provide a perspective on the multifaceted nature of consciousness research, drawing on scientists from various cognitive science disciplines. In addition to existing theories, many new conceptualizations have been proposed in light of recent advancements and empirical evidence in the field (Andersen; Fink; Gallagher; Josipovic; Tsuchiya et al.), some of which incorporate conceptualizations of AI (Mogi; Prentner and Hoffman). Several methodological proposals have been developed to assess existing theories (Kirkeby-Hinrup; Ruan). Importantly, the ongoing debate on consciousness also has significant implications for clinical research and practice (Gray Hardcastle; Oblak et al.; Pehlivanova et al.). Insights from consciousness research encompass diverse themes and approaches, offering a complex perspective on the fascinating and intriguing phenomenon of consciousness and its many facets.
